# Parenchymal and Functional Lung Changes after Stereotactic Body Radiotherapy for Early-Stage Non-Small Cell Lung Cancer—Experiences from a Single Institution

**DOI:** 10.3389/fonc.2017.00215

**Published:** 2017-09-19

**Authors:** Juliane Hörner-Rieber, Julian Dern, Denise Bernhardt, Laila König, Sebastian Adeberg, Vivek Verma, Angela Paul, Jutta Kappes, Hans Hoffmann, Juergen Debus, Claus P. Heussel, Stefan Rieken

**Affiliations:** ^1^Department of Radiation Oncology, University Hospital Heidelberg, Heidelberg, Germany; ^2^Heidelberg Institute of Radiation Oncology, Heidelberg, Germany; ^3^University of Nebraska Medical Center, Department of Radiation Oncology, Nebraska Medical Center, Omaha, NE, United States; ^4^Department of Pneumology, Thoraxklinik, Heidelberg University, Heidelberg, Germany; ^5^Translational Research Unit, Thoraxklinik, Heidelberg University, Germany Translational Lung Research Centre Heidelberg (TLRC-H), German Centre for Lung Research (DZL), Heidelberg, Germany; ^6^Department of Thoracic Surgery, Thoraxklinik, Heidelberg University, Heidelberg, Germany; ^7^Department of Diagnostic and Interventional Radiology, University-Hospital, Heidelberg, Germany; ^8^Department of Diagnostic and Interventional Radiology with Nuclear Medicine, Thoraxklinik at University-Hospital, Heidelberg, Germany

**Keywords:** non-small cell lung cancer, stereotactic body radiotherapy, radiation pneumonitis, radiation fibrosis, pulmonary function, lung injury

## Abstract

**Introduction:**

This study aimed to evaluate parenchymal and functional lung changes following stereotactic body radiotherapy (SBRT) for early-stage non-small cell lung cancer (NSCLC) patients and to correlate radiological and functional findings with patient and treatment characteristics as well as survival.

**Materials and methods:**

Seventy patients with early-stage NSCLC treated with SBRT from 2004 to 2015 with more than 1 year of CT follow-up scans were analyzed. Incidence, morphology, severity of acute and late lung abnormalities as well as pulmonary function changes were evaluated and correlated with outcome.

**Results:**

Median follow-up time was 32.2 months with 2-year overall survival (OS) of 83% and local progression-free survival of 88%, respectively. Regarding parenchymal changes, most patients only developed mild to moderate CT abnormalities. Mean ipsilateral lung dose (MLD) in biological effective dose and planning target volume size were significantly associated with maximum severity score of parenchymal changes (*p* = 0.014, *p* < 0.001). Furthermore, both maximum severity score and MLD were significantly connected with OS in univariate analysis (*p* = 0.043, *p* = 0.025). For functional lung changes, we detected significantly reduced total lung capacity, forced expiratory volume in 1 s, and forced vital capacity (FVC) parameters after SBRT (*p* ≤ 0.001). Multivariate analyses revealed SBRT with an MLD ≥ 9.72 Gy and FVC reduction ≥0.54 L as independent prognostic factors for inferior OS (*p* = 0.029, *p* = 0.004).

**Conclusion:**

SBRT was generally tolerated well with only mild toxicity. For evaluating the possible prognostic impact of MLD and FVC reduction on survival detected in this analysis, larger prospective studies are truly needed.

## Introduction

Stereotactic body radiotherapy (SBRT) is the standard of care for medically inoperable, early-stage non-small cell lung cancer (NSCLC) patients (1–3). After the introduction of SBRT, substantially higher overall survival (OS) rates were reported for this patient group by three large population-based analyses from the Netherlands and the US (4–6). Several prospective studies then demonstrated excellent 3-year local control around 90%, and survival rates of more than 50% in a highly comorbid population (7–9). SBRT has hence become the most optimal treatment option for patients with highly reduced pulmonary function [forced expiratory volume in 1 s (FEV_1_) < 30%] suffering from severe chronic obstructive pulmonary disease (COPD) (GOLD III–IV) (10, 11).

However, despite the high conformality of SBRT, toxicities are a non-trivial result of SBRT, especially in a population with poor pulmonary function. Depending on the study, symptomatic radiation pneumonitis occurs in about 10–30% of patients, which can impair patient quality of life (12–15). Fatal (grade 5) radiation pneumonitis following SBRT is only reported in very rare cases (15–17). An understudied aspect of adverse effects is the association with parenchymal remodeling following SBRT, which is detected to some degree in nearly all patients (18). Fibrosis in the high-dose regions is found in about 80% of patients and can make for a challenging differentiation between benign radiologic changes and local recurrence (18, 19).

Whether post-SBRT lung scarring correlates with significant clinical changes in pulmonary function is controversial (20, 21). Stone et al. recently showed significant impairment in pulmonary function after SBRT in a prospective trial, but though this was not associated with worse OS (22). On the basis of these results, we conducted a toxicity analysis examining post-SBRT parenchymal and functional changes, and the influence on outcome in patients who are deemed inoperable due to medical comorbidities.

## Materials and Methods

### Patient Population

Seventy consecutive patients treated between February 2004 and May 2015 were chosen for this analysis. Inclusion criteria were as follows: (1) receipt of SBRT for medically inoperable early-stage NSCLC (cT1-3cN0cM0) and (2) regular follow-up CT scans for at least 1 year at the Department of Radiation Oncology at the University Hospital Heidelberg, the Thoraxklinik Heidelberg, or at the German Cancer Research Center. The study and the study protocol were reviewed and approved by the Ethics committee of the University Hospital Heidelberg (S-140/2016). According to the decision of the Ethics committee, obtaining of written informed consent was not necessary due to the retrospective character of the study. Patients included in the analysis were identified from our cancer database. Anonymized patient data were used for analysis.

### Treatment Characteristics

Patient selection, imaging protocols, and detailed treatment techniques have been reported previously (23–25). Risk-adapted fractionation schemes were used, meaning that dose and fractionation schemes were adjusted based on tumor size and location (peripheral vs. central). Until 2011, patients were generally treated with a single fraction of 20–24 Gy prescribed to the 80% isodose line, depending on proximity to critical structures (*n* = 32). Thereafter, peripheral lesions were irradiated with three fractions of 15–18 Gy, prescribed to the conformally enclosing 65% isodose line, while central lesions received eight fractions of 7.5 Gy prescribed to the 80% isodose line (*n* = 38). Delivery techniques were 3-D (*n* = 49), helical TomoTherapy^®^ (*n* = 11), and volumetric-modulated arc therapy (*n* = 10).

### Outcome Evaluation

Routine follow-up visits involved a contrast-enhanced CT scan of the thorax around the 3-, 6-, and 12-month intervals following SBRT. If no tumor recurrence was detected in the CT scan after 12 months, CTs and X-rays were done alternately every 6–12 months thereafter. Patients with reduced performance scores often only received X-rays after 12 months. Local progression referred to progression of the tumor within the high-dose volume. Differentiation between local progression and benign fibrosis in the high-dose volume is known to be challenging. Positron emission tomography (PET)-CT scans or biopsy was used to distinguish between benign lesions and tumor recurrence.

To correlate irradiated doses with clinical results, biological effective doses (BEDs) were calculated: an α/β ratio of 10 and 3 Gy was assumed for the tumor and lung tissue, respectively. BED was calculated using the linear-quadratic model:

$$\text{BED }\left(\text{Gy}\right)=\text{fractional dose}\times \text{number of fractions }\left(1+\frac{\text{fractional dose}}{\alpha /\beta }\right).$$

Pulmonary function tests (PFTs) of all patients as performed 1–0 month before SBRT and in median 9.3 months after SBRT (5.8–18.1 months) were analyzed. These included the following: FEV_1_, forced vital capacity (FVC), total lung capacity (TLC), residual volume, and airway resistance (R).

**Figure 1:**
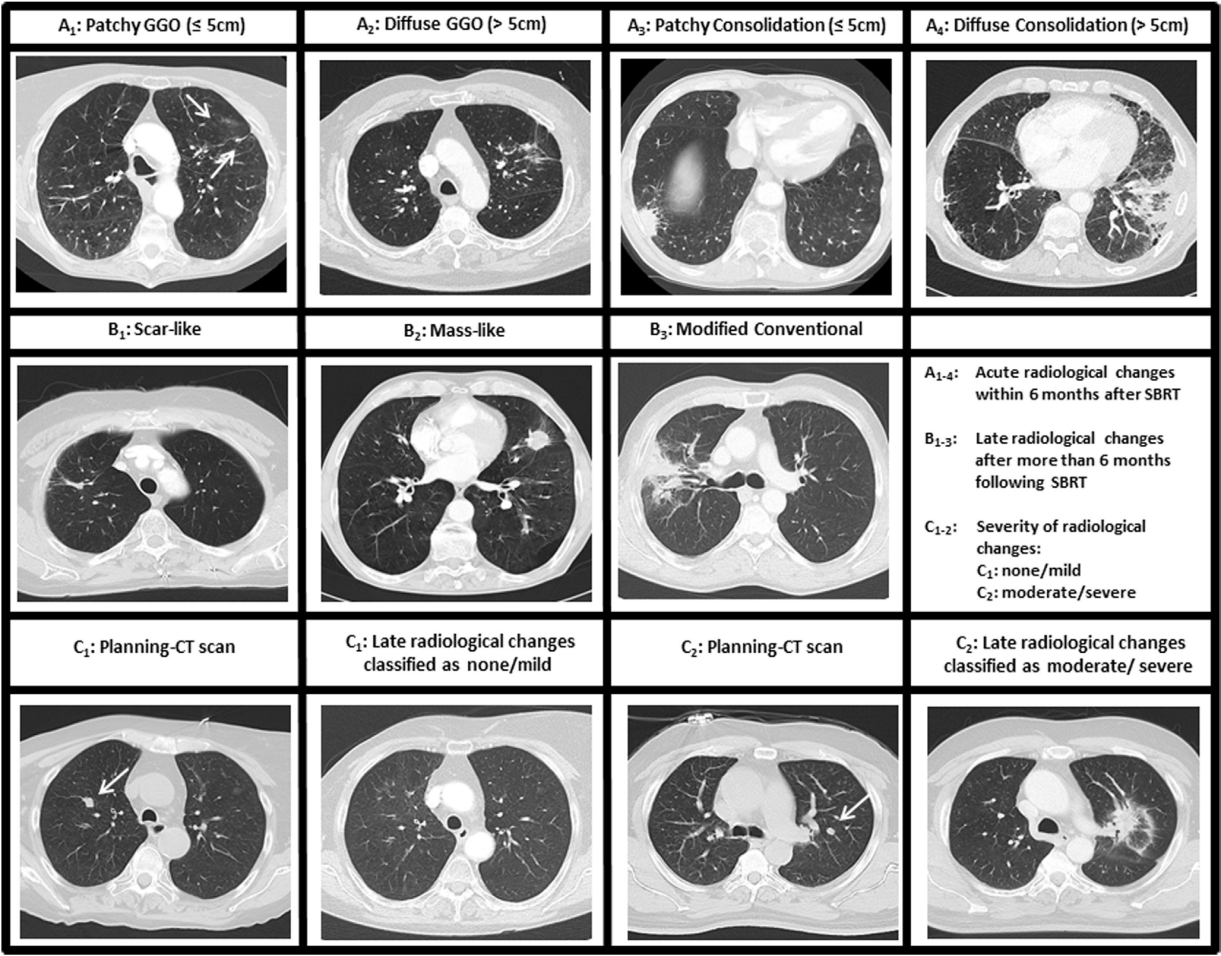
Classification of radiologic changes following stereotactic body radiotherapy (SBRT). **(A_1–4_)** Acute parenchymal changes within the first 6 months after SBRT. One category [no parenchymal abnormalities (NPA)] is not shown. **(B_1–3_)** Late parenchymal changes after 6 months following SBRT. One category (NPA) is not shown. **(C_1–2_)** Severity score for radiologic changes with **(C_1_)** classified as no/mild changes and **(C_2_)** classified as moderate/severe changes. GGO, ground-glass opacity.

Radiologic changes were defined as acute changes when they were registered within the first 6 months following SBRT, and late changes when they occurred at or after 6 months. The applied classification system was initially described by Trovo et al. and later specified by Dahele et al. (18, 26). Herein, acute findings were grouped into five categories: no parenchymal abnormalities (NPA), patchy ground-glass opacity (PGGO), diffuse GGO (DGGO), patchy consolidation (PCO), and diffuse consolidation (DCO) (Figure 1A). Late CT changes were classified into four different categories: NPA, scar-like fibrosis (SLF), mass-like fibrosis (MLF), and modified conventional pattern of fibrosis (MCPF) (Figure 1B) (18, 26). Radiologic changes were categorized by two experienced radiation oncologists with the support of an experienced pulmonary radiologist.

For general scoring, the severity score that was introduced by Dahele et al. was applied. Radiographic changes were classified as “severe” (massive changes), “moderate” (extensive, but commonly expected changes), “mild/minor” (rare changes only), or “none” (Figure 1C).

### Statistical Analysis

Overall survival, local progression-free survival (LPFS), and distant progression-free survival (DPFS) were calculated using the Kaplan–Meier method. Survival curves were compared between groups in a univariate analysis applying the log-rank test or Cox regression analysis. Multivariable Cox models were performed including all variables with *p* ≤ 0.05 in univariate analysis. Correlations between baseline factors as well as irradiation doses and severity of CT changes were assessed using Spearman’s or Pearson’s correlation coefficients. McNemar’s test was applied to calculate the association between early and late severity scores. Descriptive statistics were performed by using Mann–Whitney *U* tests or χ^2^ tests for continuous or categorical data, respectively. The non-parametric Wilcoxon signed-rank test was applied for assessing pulmonary function changes. Receiver operating characteristics (ROC) curves and the Youden’s index were performed to determine the optimal cutoff for FVC reduction or mean ipsilateral lung dose in BED (MLD) in predicting OS after 2 years. A *p*-value ≤ 0.05 was considered statistically significant. All statistical analyses were performed with SPSS software (version 20.0).

## Results

### Survival and Local Control

**Figure 2:**
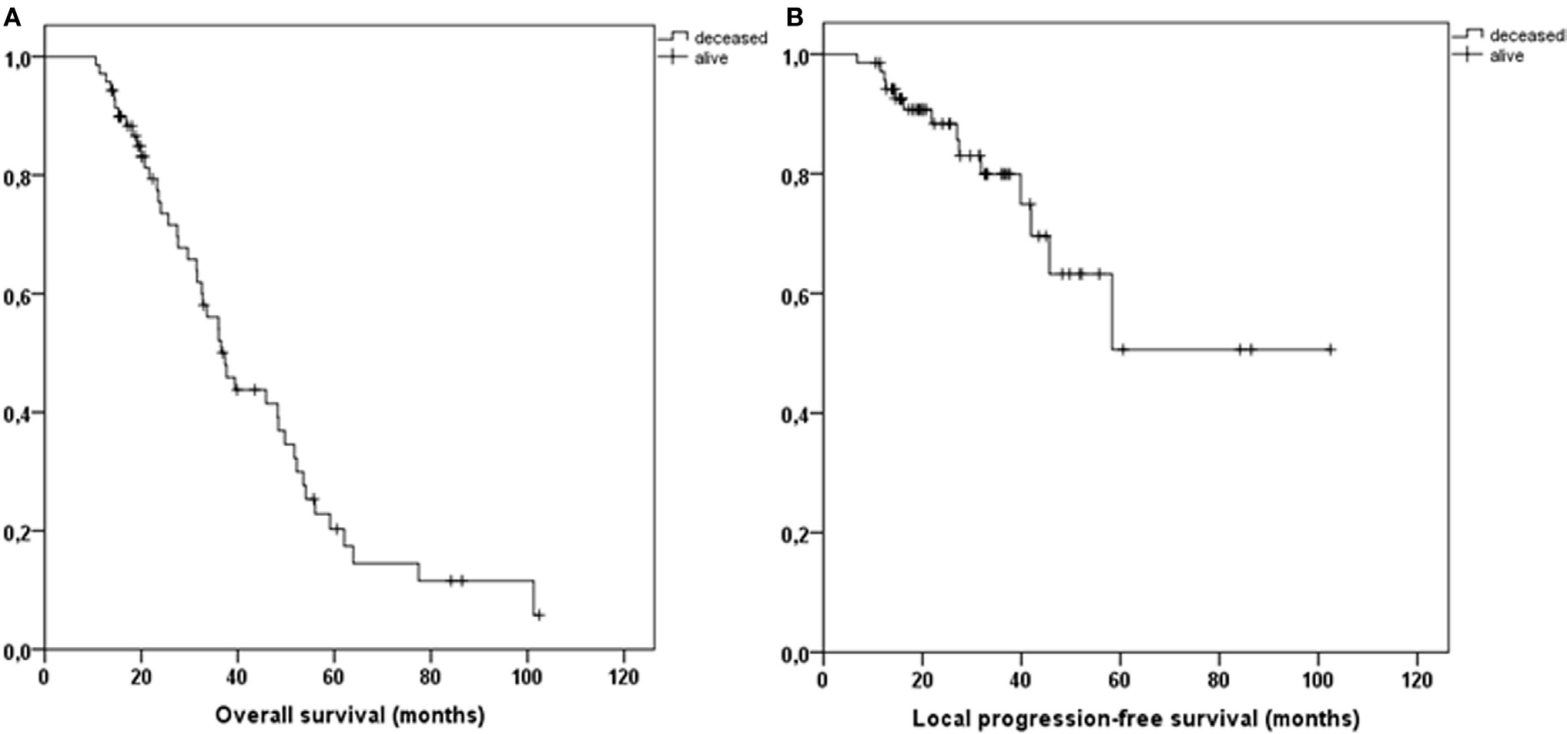
Kaplan–Meier curves illustrating overall survival **(A)** and local progression-free survival **(B)** for all patients.

Patient and treatment characteristics are displayed in Table 1. With a median follow-up time of 32.2 months (range 14.6–104.3 months), 2- and 3-year OS was 83% and 60%, respectively (Figure 2A). Two- and three-year LPFS was 88% and 80% (Figure 2B), while 2- and 3-year DPFS was, respectively, 84% and 74%. OS, LPFS, and DPFS were not significantly affected by any potential risk factor investigated (Table 2).

**Table 1:** Patient and treatment characteristics.

Patients	
Sex	
Male	50 (71.4%)
Female	20 (28.6%)
Median age (range)	70.8 years (56.5–90.4)
≥70 years	43 (61.4%)
<70 years	27 (38.6%)
Median Karnofsky performance score (range)	65% (40–80)
Staging FDG-PET	
Yes	47 (67.1%)
No	23 (32.9%)
Histology	
Adenocarcinoma	29 (41.4%)
Squamous cell carcinoma	17 (24.3%)
Others	17 (24.3%)
No histological confirmation	7 (10.0%)
TNM stage	
Stage Ia	42 (60.0%)
Stage Ib	26 (37.1%)
Stage IIa	0 (0%)
Stage IIb	2 (2.9%)
Tumor location	
Peripheral	58 (82.9%)
Central	12 (17.1%)
Smoking status	
Active smokers	22 (31.4%)
Former smokers	42 (60.0%)
Never smokers	1 (1.4%)
Smoking status not known	5 (7.2%)
Median packyears	40 pys (5–120)
Median total dose in BED (PTV encompassing)	105.0 Gy (60–151.2)
Median PTV-encompassing single dose	18.0 Gy (7.5–24.0)
Median number of fractions	3 (1–8)
Median PTV size (range)	52.0 ml (5.9–169.1)
Median ipsilateral lung dose in BED	8.31 Gy (0.62–32.5)

*SBRT, stereotactic body radiotherapy; FDG-PET, fluoro-deoxy-glucose positron emission tomography; BED, biological effective dose; PTV, planning target volume*.

**Table 2:** Univariate analysis of overall survival (OS), local progression-free survival (LPFS), and distant progression-free survival (DPFS).

Factor	*p*-Value		
	OS	LPFS	DPFS
Sex	0.837	0.701	0.716
Male			
Female			
Age	0.382	0.930	0.276
Karnofsky performance score	0.600	0.318	0.674
Staging FDG-PET	0.494	0.085	0.824
Yes			
No			
Histology	0.950	0.339	0.245
Adenocarcinoma			
Squamous cell carcinoma			
Others			
No histological confirmation			
TNM stage	0.608	0.671	0.656
Stage Ia			
Stage Ib			
Stage IIa			
Stage IIb			
Tumor location	0.369	0.196	0.115
Peripheral			
Central			
Smoking status	0.491	0.512	0.674
Active smokers			
Former smokers			
Never smokers			
Smoking status not known			
Median packyears	0.734	0.764	0.222
Total dose in BED (PTV encompassing)	0.854	0.395	0.522
BED ≥ 100 Gy			
BED < 100 Gy			
PTV-encompassing single dose	0.696	0.380	0.781
Number of fractions	0.407	0.419	0.823
PTV size	0.408	0.675	0.324

*FDG-PET, fluoro-deoxy-glucose positron emission tomography; BED, biological effective dose; PTV, planning target volume*.

*The variables sex, staging FDG-PET, histology, TNM stage, tumor location, smoking status, and PTV-encompassing biological effective total dose were analyzed as categorical variables, while the other variables were taken as continuous variables for analysis*.

### Parenchymal Lung Changes after SBRT

In total, 463 CT scans of 70 patients were reviewed for parenchymal lung changes. A median of five CT scans (range 3–17) could be evaluated per patient covering a time frame of in median 20.0 months after SBRT (range 12.2–78.8 months). The median time to onset of CT changes was 2.5 months (range 1.6–8.8 months).

Acute radiologic changes within the first 6 months (113 CT scans available) following SBRT were assessed for each patient: NPA were detected in 10% of the cases, while 63 patients (90%) displayed acute parenchymal changes. From this cohort, 11% PGGO, 25% DGGO, 25% PCO, and 29% DCO (Figure 1A).

Late parenchymal changes were detected to some degree in all CT scans available (Figure 1B). After 6 months following SBRT (60 patients with CT scans available), 10% of the cases showed SLF, 7% MLF, and in 83% of the patients MCPF was detected. Parenchymal changes slightly decreased 12 months post-SBRT with 14% SLF, 9% MLF, and 77% MCPF (64 patients with CT scans available). After 18 months, a further reduction in parenchymal changes was registered (156 CT scans in 41 patients): 20% SLF, 9% MLF, and 71% MCPF.

Most of the tumors had an acute severity score of 0 (none, *n* = 10, 14%), 1 (mild, *n* = 43, 62%), or 2 (moderate, *n* = 16, 23%). Only one patient each suffered from acute severe changes (score = 3) after SBRT. The pattern for chronic severity score was as follows: mild (score 1): 66%, moderate (score 2): 33%, and severe (score 1): 1%. The two patients with severe radiologic changes developed radiation pneumonitis CTCAE grade III requiring corticosteroids and oxygen support until resolution of symptoms. Two additional patients developed CTCAE grade II radiation pneumonitis. In total, 5.7% of the patients suffered from grade ≥II radiation pneumonitis.

The severity of acute CT changes predicted for those of late changes (*p* = 0.027). We did not detect any significant correlations between maximum severity score for each tumor and gender (*p* = 0.085), patient age (*p* = 0.366), Karnofsky performance score (*p* = 0.426), tumor histology (*p* = 0.333), TNM stage (*p* = 0.190), tumor location (*p* = 0.329), smoking status (*p* = 0.502), smoking history in number of packyears (*p* = 0.473), total dose in BED (*p* = 0.705), single dose (*p* = 0.643), and number of fractions (*p* = 0.625). However, both planning target volume (PTV) size and MLD in BED were predictive for parenchymal lung changes measured as the maximum severity score (respectively, *p* < 0.001 and *p* = 0.014).

**Figure 3:**
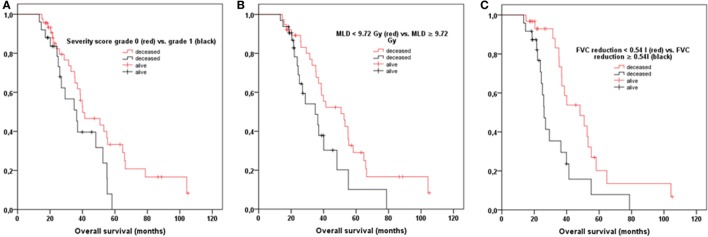
**(A)** Overall survival (OS) was significantly reduced if patients showed moderate/severe radiologic changes following stereotactic body radiotherapy (SBRT) compared to patients with only none/mild parenchymal changes (*p* = 0.043). **(B)** Patients with treated with an MLD ≥ 9.72 Gy suffered from worse OS (*p* = 0.042). **(C)** OS was significantly impaired if patients had an absolute reduction in FVC ≥ 0.54 L following SBRT (*p* = 0.007). FVC, forced vital capacity; MLD, mean ipsilateral lung dose in biological effective dose.

Furthermore, OS was significantly reduced if scans showed moderate or severe parenchymal lung changes (*p* = 0.043, HR 1.928 [CI 1.020–3.644]) (Figure 3A). Specifically, patients with a maximum severity score of 0–1 (none/mild) showed 2- and 3-year OS of 83 and 65%, while patients with a maximum severity score of 2–3 (moderate/severe) experienced 2- and 3-year OS of 78 and 51%, respectively (*p* = 0.043, HR 1.928 [CI 1.020–3.644]). In addition, OS was significantly influenced by MLD but not by PTV size (*p* = 0.025, HR 1.046 [CI 1.002–1.092]; *p* = 0.408, HR 1.004 [CI 0.995–1.013]) (Figure 3B). A cutoff MLD of 9.72 Gy was calculated in ROC analysis. Hence, patients treated with an MLD < 9.72 Gy showed 2- and 3-year OS of 89.2% and 67.7%, while patients with an MLD ≥ 9.72 Gy only had 2- and 3-year OS rates of 73.6% and 48.6%, respectively (*p* = 0.042; 1.904 [CI 1.017–3.563]). Both LPFS and DPFS were not significantly affected by maximum severity score, MLD, or PTV size (*p* ≥ 0.05).

### Functional Lung Changes after SBRT

In total, paired PFTs were available for 57 of the analyzed 70 patients before and after SBRT. PFTs were obtained at a median of 44 days before SBRT (range 3–70 days) and 9.3 months (range 5.8–18.1 months) after SBRT. Detailed PFT data are illustrated in Table 3.

**Table 3:** Mean pulmonary function tests (PFTs) for 58 patients before and after SBRT.

PFT parameter (range)	Before SBRT	After SBRT	Absolute difference	Relative difference (%)	*p*-Value
TLC	6.96 l (3.77–11.34)	6.44 l (3.10–12.51)	−0.52 l (−3.10 to +2.20)	−7.50 (−28.5 to +12.8)	0.001*
FVC	2.81 (1.19–4.30)	2.36 (1.09–3.85)	−0.45 l (−1.36 to +1.10)	−16.0 (−34.1 to +5.4)	<0.001*
FEV_1_	1.51 l (0.50–2.93)	1.36 l (0.50–2.84)	−0.17 l (−0.79 to +0.52)	−9.8 (−33.9 to +33.3)	<0.001*
FEV_1%_ predicted	57.5% (25.3–89.9)	52.29% (−24.2 to 90.3)	−5.18% (−28.2 to +4.8)		<0.001*
FEV_1_/FVC	52.1% (21.4–81.5)	41.8% (22.2–89.1)	−10.3% (−21.6 to +23.1)		0.103
Air way resistance	0.49 kPa s/l (0.09–1.80)	0.58 kPa s/l (0.13–1.49)	+0.09 kPa s/l (−0.81 to +0.87)	+18.4 (+90.0 to −30.6)	0.003*

*SBRT, stereotactic body radiotherapy; TLC, total lung capacity; FVC, forced vital capacity; FEV_1_, forced expiratory volume in 1 s; FEV_1_/FVC, forced expiratory volume in 1 s divided by forced vital capacity*.

*p ≤ 0.05

All analyzed baseline pre- and posttreatment PFT parameters did not significantly affect OS, LPFS, and DPFS (*p* > 0.05). However, SBRT treatment significantly reduced post-SBRT lung function: TLC (−0.52 L; *p* = 0.001), FVC (−0.45 L, *p* < 0.001), FEV_1_ (−0.17 L, *p* < 0.001), FEV_1%_ (−5.2%, *p* < 0.001), and airway resistance (+0.09 kPa s/L, *p* = 0.003) (Table 3).

As a next step, we evaluated whether absolute differences between pre- and post-interventional PFT parameters could predict outcome. While we did not detect a significant effect of TLC, FEV_1_, FEV_1%_, and resistance, treatment-related reduction in FVC significantly affected survival (*p* = 0.007, 3.910 [CI 1.445–10.575]). A cutoff FVC reduction of 0.54 L was calculated in ROC analysis. Patients with a reduction in FVC ≥ 0.54 L showed significantly worse 2- and 3-year OS of 71% and 35%, while patients with an FVC reduction <0.54 L had 2- and 3-year OS rates of 93% and 73%, respectively (*p* = 0.011, 2.439 [CI 1.227–4.849]) (Figure 3C). Absolute reductions in FVC did not significantly correlate with MLD (*p* = 0.913), PTV size (*p* = 0.334), and maximum severity score of parenchymal changes (*p* = 0.546).

Finally, we performed multivariate analysis revealing MLD ≥ 9.72 Gy and FVC reduction ≥0.54 L to be statistically significant independent prognostic factors for OS (*p* = 0.029, 1.037 [CI 1.011–1.089]; *p* = 0.004, 2.347 [CI 1.167–4.723]). Maximum severity score of parenchymal changes was not identified as an independent prognostic factor for OS (*p* = 0.140, 1.289 [CI 0.601–2.766]).

## Discussion

Pulmonary SBRT is believed to be a milder way of treatment with less side effects compared to surgery involving lobectomy and systematic lymphadenectomy as it is primarily offered to patients with reduced performance score who are classified medically inoperable (2, 11, 27). In this study, we investigated early and late radiographic lung injury as well as pulmonary function changes following SBRT. In general, most patients only showed mild to moderate parenchymal and functional lung alterations that did not translate into reduced clinical performance in the majority of cases.

Regarding parenchymal lung changes in follow-up imaging studies, nearly all patients only developed minor changes, while severe changes were only noticed in two patients (2.9%) and were transient. All patients were diagnosed with radiological changes following SBRT at some time of follow-up, which is known to impair diagnosis of local recurrence (19, 28). Similar acute and chronic patterns of CT changes were reported by Trovo et al. and Dahele et al. (18, 26).

However, to the best of our knowledge, this is the first investigation describing a significant association between MLD and survival following SBRT (Figures 3A,B).

Several groups have shown a dose–response relationship for radiation-induced pneumonitis following SBRT (29, 30). Furthermore, a recent pooled analysis of 88 studies investigating lung toxicity after SBRT reported MLD as well as large tumor size to be significant adverse risk factors for pneumonitis and lung fibrosis (15). Indeed, we also detected a significant correlation between both MLD as well as PTV size and maximum severity score of radiological CT changes. In comparison, when regarding radiotherapy for locally advanced NSCLC patients, an association between radiation exposure to normal lung and severe pneumonitis is also well known (31). Furthermore, development of radiation pneumonitis and generalized radiological changes after radiotherapy in locally advanced NSCLC patients were shown to be independent negative prognostic factors for survival (32). A recent study even underlined the predictive impact of lung dose and especially MLD on survival analyzing prognostic factors in 468 patients with stage IIIA–IIIB NSCLC (33). Our study now shows that SBRT with an MLD ≥ 9.72 Gy was associated with significantly worse survival. However, due to the low number of patients and the limited number of events recorded in this analysis, this finding has to be interpreted with caution. All patients included in this study were classified medically inoperable and suffered from severe pulmonary comorbidities, which probably highly impaired survival. Nevertheless, a recent study showed that dose to heart substructures was associated with non-cancer death after SBRT in stage I–II NSCLC patients (34). Hence, dose spillage to the heart and healthy lung tissue should be kept as low as reasonably possible when performing SBRT.

In a second step, we analyzed functional lung changes and detected a significant decline following SBRT for TLC, FVC, FEV_1_, and FEV_1%_ (*p* ≤ 0.001). In contrast to our results, Stanic et al. and Stephans et al. did not show any significant change in pulmonary function examining lung function in 55 and 92 patients after SBRT (21, 35). However, a recent study by Stone et al. also reported a significant decline for FEV_1_, diffusion capacity, FVC, and TLC following SBRT (22). Nevertheless, most studies did not show any association of lower baseline or post-SBRT pulmonary function with worse survival (21, 22, 36, 37). Guckenberger et al. only described a significant impact of pretreatment, and not posttreatment, diffusion capacity of carbon monoxide on survival (thus not treatment-related) (10). Notably, absolute reduction in FVC was shown to be an independent prognostic factor for OS in this analysis (Figure 3C), indicating a possible influence of radiation-induced restrictive lung disease upon survival. This might be due to the fact that this analysis is the only study investigating the prognostic impact of absolute loss in PFTs and not only pre- and posttreatment pulmonary function parameters.

Similar to other studies reduction in FVC did not significantly correlate with prognostic factors for lung toxicity such as MLD, and PTV size in this analysis (20, 21). There was no significant correlation between absolute reduction in FVC and maximum severity score of parenchymal changes. This finding is supported by reports about conventionally fractionated radiotherapy in which a dose–effect relationship for posttreatment PFT changes is also missing (38). This might be explained by the fact that irradiation of lung tumors may even improve PFT by tumor shrinkage or reopening of atelectasis (39). Furthermore, the vast majority of these patients had severe COPD with decline in pulmonary function on the basis of natural disease progression (40, 41). Hence, the detected loss in pulmonary function has to be interpreted with caution and might also be caused by the natural progression of preexisting COPD (40, 41). Larger, multicenter studies are truly needed to evaluate the possible prognostic impact of MLD and lung function changes following SBRT on survival. In this study, we did not detect distinct factors for surely predicting possible lung toxicity following SBRT. Nevertheless, other factors such as pretreatment immune status are reported to predict for toxicity after SBRT (42).

Despite the reported parenchymal and functional lung changes, survival and local control rates detected in this analysis were comparable to other studies and still much higher in comparison to conventionally fractionated radiotherapy (7–9, 16, 43). In detail, 3-year OS, progression-free survival (PFS), LPFS, and DPFS rates were 60, 65, 80, and 74%, respectively. Regarding current guidelines, a benchmark is the 3-year local rate following SBRT which is supposed to be 90% and higher when a BED > 100 Gy is applied (2). As this study included data from 2004 to 2015, several patients were treated with lower doses which might have led to the slightly reduced local control rates in this analysis.

The higher PFS compared to the detected OS in this analysis might raise the question whether some patients were overtreated, although other studies reported similar results (42, 44). Furthermore, not all patients received histopathological confirmation of disease due to reduced performance score but had fluoro-deoxy-glucose positron emission tomography positive tumors. The severity of pulmonary comorbidities is known to be an important predictor for survival for lung cancer patients—not only after SBRT (45, 46). However, two recent reports stated that withholding SBRT in patients with severe COPD is not justified (47, 48). Hence, in some patients SBRT might transfer the cause of death from tumor disease to pulmonary comorbidities.

As pulmonary SBRT for early-stage NSCLC was analyzed between 2004 and 2015 in this study, patients were mainly treated with less advanced radiation techniques while survival data were available therefore as a tradeoff. For example, regular performance of 4-D-CT scans to account for tumor motion started in 2009 in our department. Hence, larger safety margins leading to larger PTV sizes and higher MLD were needed. Today, advances in radiation planning and delivery techniques such as intensity-modulated radiotherapy (IMRT) and elaborate image guidance including gating and tracking help to further minimize the dose to normal tissue and therefore reduce side-effects. Some limitations of this study deserve mention. Aside from the smaller sample size and retrospective nature, paired PFTs were not available for all patients. Second, further lung dose parameters as *V*_5Gy_ and *V*_20Gy_ were not accessible. Third, a larger cutoff interval for follow-up CT scans of more than one year was not possible, as several patients only received X-ray scans for follow-up imaging after 1 year due to their poor performance status. Fourth, due to the retrospective character of this study, detailed analysis of cardiopulmonary comorbidities and their potential impact on survival in this study was not possible.

Analyzing parenchymal and functional lung injury following SBRT, we detected only mild radiological changes and tolerable reduction in pulmonary function for most patients. However, this study showed a significant association between SBRT with a higher MLD and inferior survival. Furthermore, higher absolute reduction in FVC significantly impaired survival in this analysis. Nevertheless, these results have to be interpreted with caution due to the limited number of patients and the retrospective character of this study. Natural progression of pulmonary comorbidities including COPD surely also led to reduced survival in this patient group. If toxicity of SBRT had an impact on survival in this study, this was potentially caused by the interaction with preexisting pulmonary comorbidities.

Based on this study, modern radiotherapy methods including delivery techniques such as IMRT and daily image guidance should be applied for minimizing PTV sizes and keeping MLD as low as reasonable possible. Furthermore, larger prospective and multicenter studies are highly needed for evaluating the potential prognostic impact of parenchymal and functional lung changes on survival.

## Ethics Statement

This retrospective study was carried out in accordance with the recommendations of the Ethics committee of the University Hospital Heidelberg. According to the committee’s decision based on the retrospective character of this analysis, no additional written informed consent of all patients was needed.

## Author Contributions

JH-R carried out data collection as well as statistical analysis and drafted the manuscript. JDern helped with data collection. DB, SA, and AP assisted with patient care. LK helped with figure and table preparation. VV assisted with manuscript preparation. JK and HH were responsible for pulmonary patient treatment. CH supported assessment of radiologic changes on CT scans and classifying of severity. SR and JDebus conceived of the study, and participated in its design and coordination and helped to draft the manuscript. All the authors were responsible for data interpretation, participated in manuscript revisions, and approved the final manuscript.

## Conflict of Interest Statement

The authors declare that the research was conducted in the absence of any commercial or financial relationships that could be construed as a potential conflict of interest.
